# Mulberry fruit prevents LPS-induced NF-κB/pERK/MAPK signals in macrophages and suppresses acute colitis and colorectal tumorigenesis in mice

**DOI:** 10.1038/srep17348

**Published:** 2015-11-30

**Authors:** Zhengjiang Qian, Zhiqin Wu, Lian Huang, Huiling Qiu, Liyan Wang, Li Li, Lijun Yao, Kang Kang, Junle Qu, Yonghou Wu, Jun Luo, Johnson J. Liu, Yi Yang, Wancai Yang, Deming Gou

**Affiliations:** 1 Shenzhen Key Laboratory of Microbial Genetic Engineering, College of Life Sciences, Shenzhen University, Shenzhen, Guangdong, 518060, China; 2 Key Laboratory of Optoelectronic Devices and Systems of Ministry of Education and Guangdong Province, College of Optoelectronic Engineering, Shenzhen University, Shenzhen, Guangdong, 518060, China; 3 School of Basic Medical Sciences, Shenzhen University, Shenzhen, Guangdong, 518000, China; 4 College of Animal Science and Technology, Northwest A&F University,Yangling, 712100, Shaanxi, China; 5 Department of Pharmacology, School of Medical Sciences, Faculty of Medicine, University of New South Wales, Sydney, NSW 2052 Australia; 6 Department of Pathology and Institute of Precision Medicine, Jining Medical University, Jining, Shandong, 272067, China

## Abstract

Here, we investigated the impact of mulberry fruit (MBF) extracts on lipopolysaccharide (LPS)-induced inflammatory responses in RAW 264.7 macrophages, and the therapeutic efficacy of MBF diet in mice with dextran sulfate sodium (DSS)-induced acute colitis and MUC2^−/−^ mice with colorectal cancer. *In vitro*, LPS-induced nitric oxide (NO) production was significantly inhibited by MBF extracts via suppressing the expression of proinflammatory molecules, including inducible nitric oxide synthase (iNOS), cyclooxygenase-2 (COX-2), interleukin-1 beta (IL-β) and IL-6. Particularly, a dose-dependent inhibition on LPS-induced inflammatory responses was observed following treatment with MBF dichloromethane extract (MBF-DE), in which linoleic acid and ethyl linolenate were identified as two active compounds. Moreover, we elucidated that MBF-DE attenuated LPS-induced inflammatory responses by blocking activation of both NF-κB/p65 and pERK/MAPK pathways. *In vivo*, DSS-induced acute colitis was significantly ameliorated in MBF-fed mice as gauged by weight loss, colon morphology and histological damage. In addition, MBF-fed MUC2^−/−^ mice displayed significant decrease in intestinal tumor and inflammation incidence compared to control diet-fed group. Overall, our results demonstrated that MBF suppressed the development of intestinal inflammation and tumorgenesis both *in vitro* and *in vivo*, and supports the potential of MBF as a therapeutic functional food for testing in human clinical trials.

Mulberry (*Morus alba* L.), a genus of the *Moraceae* family, is widely cultivated in many regions of the world, predominantly in eastern, southern and southeastern Asia^1^. Due to its chemical composition and pharmacological activity, various parts of mulberry including leaves, root barks, branches and fruits have long been used in traditional oriental medicine^2^,^3^,^4^. Among them, mulberry fruit (MBF) is commonly eaten or made into wine, fruit juice, jam and canned food^5^. Accumulative researches indicated that MBF was associated with the prevention and amelioration of numerous chronic diseases^6^,^7^,^8^,^9^. For instance, MBF ethyl acetate-soluble extract was shown to have crucial anti-diabetic and antioxidant activities in streptozotocin induced diabetic mice^10^. By combining treatment with mulberry leaf and fruit extracts, Lim *et al*. found significant anti-obesity and anti-inflammatory activities in high-fat diet-induced obese mice^11^. These beneficial effects of MBF are putatively attributable to bioactive ingredients such as alkaloids, polyohenol, flavonoids anthocyanins and carotenoids^10^,^12^,^13^,^14^. Despite of the promising results with the use of MBF in the management of many diseases, the bioactive components and biofunctional activity of MBF remain to be explored.

Inflammatory bowel disease (IBD), of which ulcerative colitis and Crohn’s disease are two prevailing entities, is characterized by the chronic inflammation of at least part of the gastrointestinal tract^15^,^16^. The most common symptoms of IBD are episodes of bloody diarrhea, abdominal discomfort, fever and weight loss^17^,^18^. The etiology of the disease is not fully understood, but is believed to be caused by a combination of environmental, genetic and immunoregulatory factors^17^,^18^,^19^. In the early stage of IBD, a number of proinflammatory molecules such as inducible nitric oxide synthase (iNOS), cyclooxygenase-2 (COX-2), interleukin-4 (IL-4), IL6, IL-1β and tumor necrosis factor-alpha (TNF-α), have been implicated to play crucial roles in mediating immune inflammatory responses^16^. Recent advances in the understanding of pathogenic pathways of IBD have accelerated greatly the discovery of many therapeutic agents that target signaling or cascade of those proinflammatory regulators^16^,^20^.

It is now well recognized that inflammatory conditions can provide and sustain conditions favorable for tumorigenesis^21^,^22^. Specifically, IBD represents an assortment of chronic inflammatory syndromes that greatly increase the risk for developing colorectal cancer (CRC), and the risk rate is proportional to the severity, extent and duration of IBD^23^,^24^. As one of the most prevalent cancers globally, CRC has been widely studied using *in vitro* and *in vivo* experimental models, aiming to elucidate how chronic inflammation mediates the initiation and progression of CRC via its various cellular and cytokine mediators^22^,^25^,^26^. Besides that, a large number of therapeutic approaches have been adopted to prevent or treat this disease^25^. Recently, chemoprevention has received a great attention and natural medicinal plants have been recognized as important sources.

In this study, we investigated the anti-inflammatory activity of MBF extracts in lipopolysaccharide (LPS) stimulated RAW264.7 macrophage cells, and the therapeutic efficacy of MBF diet in dextran sulfate sodium (DSS) exposed mice and MUC2^−/−^ mice, which represent the acute colitis (AC) and colorectal cancer (CRC) models, respectively. Our results demonstrated that MBF extracts inhibited the expression of proinflammatory mediators by blocking both the NFκB/p65 and pERK/MAPK signals in LPS-induced macrophage cells, and the MBF diet showed significant inhibitory effects on the development of DSS-induced AC and the progress of CRC in MUC2^−/−^ mice.

## Results

### MBF extracts inhibited NO production without affecting cell viability in LPS stimulated RAW 264.7 cells

**Figure 1:**
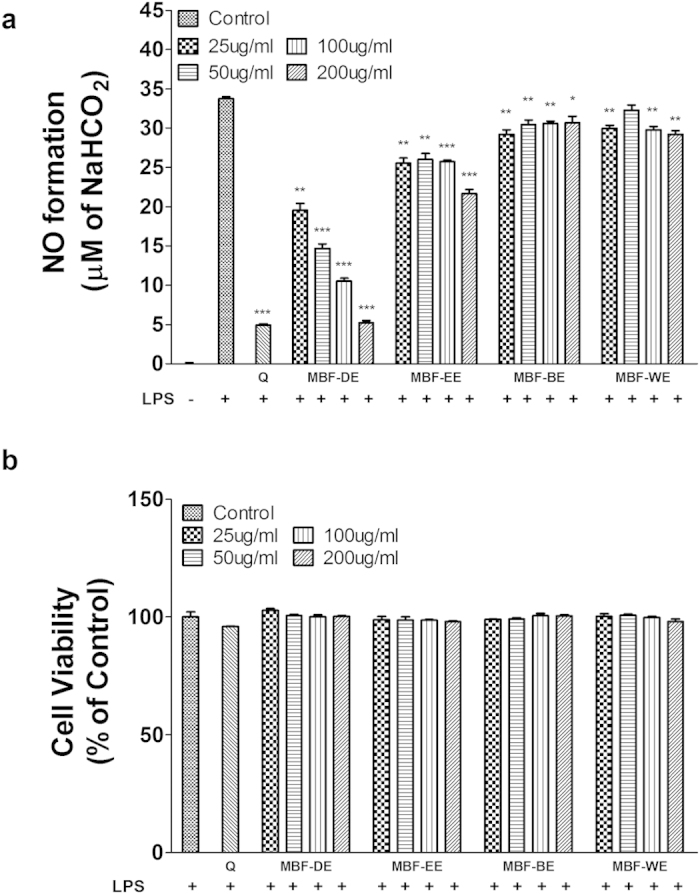
Mulberry fruit (MBF) extracts inhibit LPS-induced NO production without affecting cell viability in RAW 264.7 macrophage cells. Cells were treated with MBF dichloromethane extracts (MBF-DE), MBF ethyl acetate extracts (MBF-EE), MBF n-butanol extracts (MBF-BE) and MBF water extracts (MBF-WE) at different concentrations range from 25 to 200 μg/mL in the presence or absence of LPS (1 μg/ml) for 24 h. Quercetin (Q) was used as the positive control. NO production (**a**) and cell viability (**b**) were determined by the Griess and MTS assay, respectively. Data are expressed as means ± SD with at least three independent experiments. *P < 0.05, **P < 0.01, ***P < 0.001 compared to LPS-induced control.

After cells were treated with different concentrations of MBF dichloromethane extracts (MBF-DE), ethyl acetate extracts (MBF-EE), n-butanol extracts (MBF-BE) and water extracts (MBF-WE) ranging from 25 to 200 μg/ml in the presence of LPS, the production of nitric oxide (NO) was estimated by measuring the concentration of nitrite in cell culture medium using the Griess method. When compared with normally cultured cells in the absence of LPS, NO production was significantly induced in culture medium after 24 h of LPS treatment (Fig. 1a). However, the LPS-stimulated NO accumulation was significantly decreased by simultaneously adding MBF-DE, MBF-EE, MBF-BE or MBF-WE in the growth medium (Fig. 1a). Among them, MBF-DE treatment showed the highest inhibitory efficiency in NO production in a dose-dependent manner (Fig. 1a). However, the reduction of NO production in culture medium was not due to cytotoxicity, since cell viability was not affected by the treatment with MBF-DE, MBF-EE, MBF-BE or MBF-WE in the presence of LPS (Fig. 1b).

### MBF extracts inhibited the expression of proinflammatory mediators and cytokines in LPS stimulated RAW 264.7 cells

**Figure 2:**
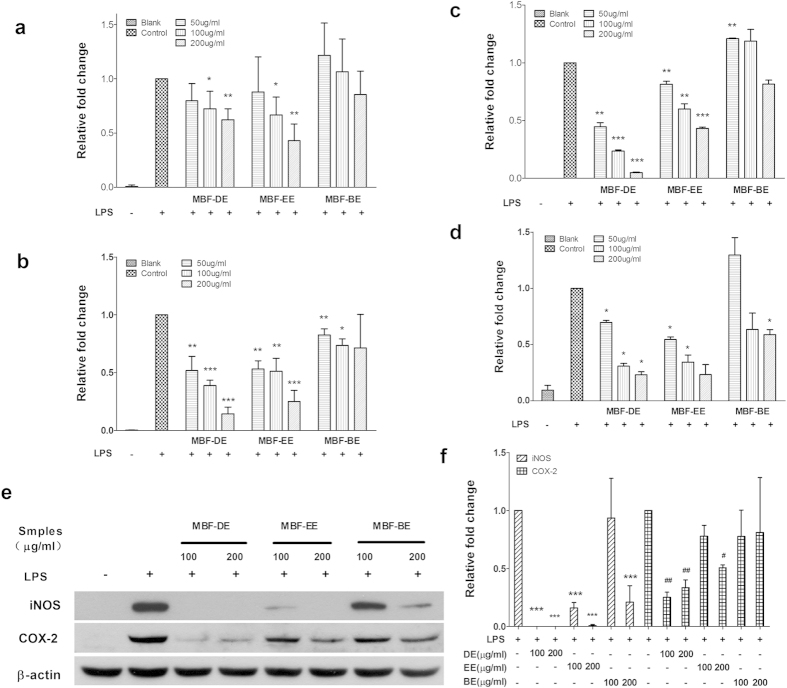
MBF extracts inhibit LPS-induced up-regulation of proinflammatory regulators in RAW 264.7 macrophage cells. Cells were stimulated for 6 h with LPS (1 μg/mL) alone or together with MBF-DE, MBF-BE and MBF-EE at concentrations indicated. The mRNA levels of iNOS (**a**), COX-2 (**b**), IL-1β (**c**) and IL-6 (**d**) were analyzed by real-time qRT-PCR and the RPL14 was used to normalize data. The protein expression of iNOS and COX-2 (**e,f**) were determined by Western blotting and β-actin was used as an internal loading control. Results are shown as means ± SD of three independent experiments. * or ^#^*P* < 0.05, ** or ^##^*P* < 0.01, ***P < 0.001 compared to LPS-induced control.

Proinflammatory mediators and cytokines such as iNOS, COX-2, IL-1β and IL-6 play important roles in regulating inflammatory response^27^,^28^. To investigate whether or not the MBF-DE, MBF-EE and MBF-BE have anti-inflammatory activities in LPS stimulated macrophages, the expression of iNOS, COX-2, IL-1β and IL-6 was measured at both transcriptional and translational levels. Compared with the blank group, LPS induced up-regulation of mRNA levels of iNOS, COX-2, IL-1β and IL-6 were markedly inhibited by the treatment with MBF-DE, MBF-EE and MBF-BE at different concentrations (Fig. 2a–d). Similar inhibitory effects of MBF extracts on protein expression of these proinflammatory genes were also found in LPS stimulated macrophages (Fig. 2e,f and Supplementary Fig. 1). Among them, a significant dose-dependent manner was observed following the treatment with MBF-DE, i.e. the higher concentration of MBF-DE applied, the greater inhibitory effect on expression of these genes were shown (Fig. 2). These results illustrated that the expression of iNOS, COX-2, IL-1β and IL-6 in LPS-stimulated macrophages were suppressed by MBF extracts, in which the MBF-DE treatment displayed a concentration-dependent inhibitory effect. Therefore, MBF-DE was used for further study in following experiments hereafter, unless stated otherwise.

### MBF-DE prevented NF-κB/p65 and MAPK/pERK signals in LPS stimulated RAW 264.7 cells

**Figure 3:**
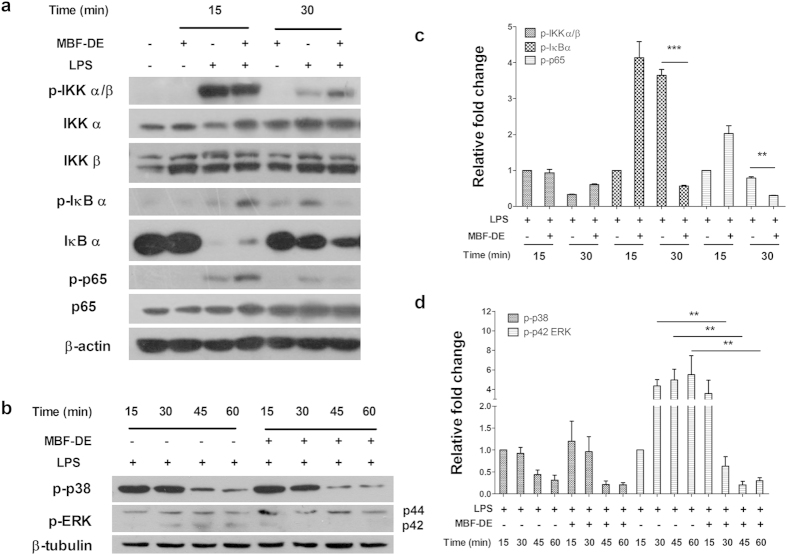
MBF-DE blocks IκBα and MAPK pERK phosphorylation in LPS-induced RAW 264.7 macrophage cells. Cells were treated with MBF-DE (200 μg/ml) in absence or presence of LPS (1 μg/ml) for the indicated time. The protein level of phospho-IKKα/β, IKKα, IKKβ, phospho-IκBα, IκBα, phospho-p65, p65, phospho-p38 and phospho-JNK were detected by Western blotting analyses using their respective antibodies. β-actin or β-tubulin was used as internal loading control. Cell culture experiments were performed at least three times. Representative results of immunoblots (**a,b**) and their quantifications (**c,d**) to better view the difference between different treatment groups were shown. Data represent means ± SEM; **P < 0.01, ***P < 0.001 compared to LPS-induced control.

The above described finding prompted us to investigate whether MBF-DE treatment was involved in NF-κB and MAPK signaling pathways, which are known to regulate the transcription of proinflammatory genes^28^,^29^,^30^. Compared to blank control, the phosphorylation status of IκBα/β, IκBα and p65 increased remarkably by LPS stimulation (Fig. 3a). However, the phosphorylation status of IκBα and p65 were significantly suppressed by the treatment with MBF-DE at 30 min of LPS stimulation (Fig. 3a,c). Concerning the MAPK pathway, a rapid decrease in phosphorylation of p38 but increase in extracellular signal-regulated kinase (ERK) was observed following LPS treatment (Fig. 3b,d). However, MBF-DE significantly blocked the change in phosphorylation status of pERK from 30 min of LPS stimulation, as compared with the same time point in the presence of LPS (Fig. 3b,d).

**Figure 4:**
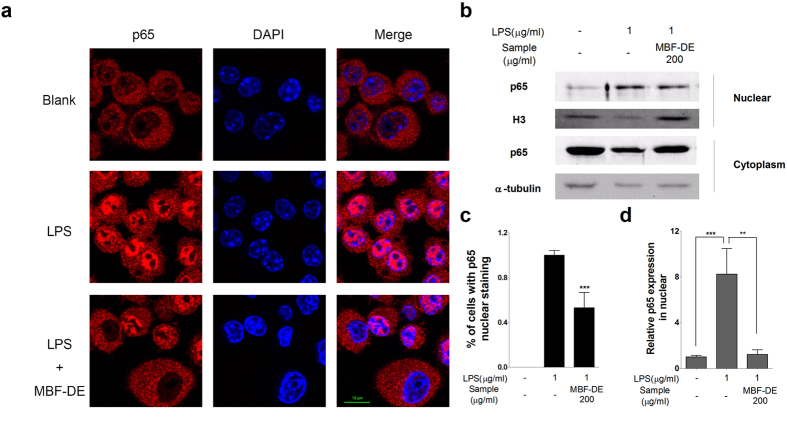
MBF-DE inhibits NF-κB p65 nuclear translocation in LPS-induced RAW 264.7 macrophage cells. Cells were pretreated with or without MBF-DE (200 μg/ml) for 12 h and then exposed to LPS (1 μg/ml) for 1 h. Then cells were fixed, permeabilized and processed using immunofluorescent staining for p65. Nuclei were stained with DAPI (**a**). The relative percentage of p65 translocation compared to LPS control was quantified based on three independent experiments (**c**). The protein level of p65 in nuclear and cytoplasmic fractions was determined by Western blotting (**b**) and the relative change was quantified (**d**). Results are shown as means ± SD of three independent experiments. **P < 0.01, ***P < 0.001 compared to LPS-induced control.

In parallel, the nuclear translocation of NF-κB/p65 was measured using confocal microscopic analysis. As shown in Fig. 4, LPS stimulation in macrophages resulted in a dramatic increase in the translocation of p65 into the nucleus, which in turn was markedly suppressed after 12 h of MBF-DE pre-treatment. This observation was further confirmed by the Western blotting, i.e., LPS induced accumulation of p65 protein in nuclear was inhibited by MBF-DE treatment (Fig. 4b,d). Taken together, these data demonstrated that MBF-DE prevented not only NF-κB signals through blocking p65 nuclear translocation and IκBα phosphorylation but also MAPK/pERK activation via its phosphorylation.

### Linoleic acid and ethyl linolenate were two active compounds in MBF-DE

**Figure 5:**
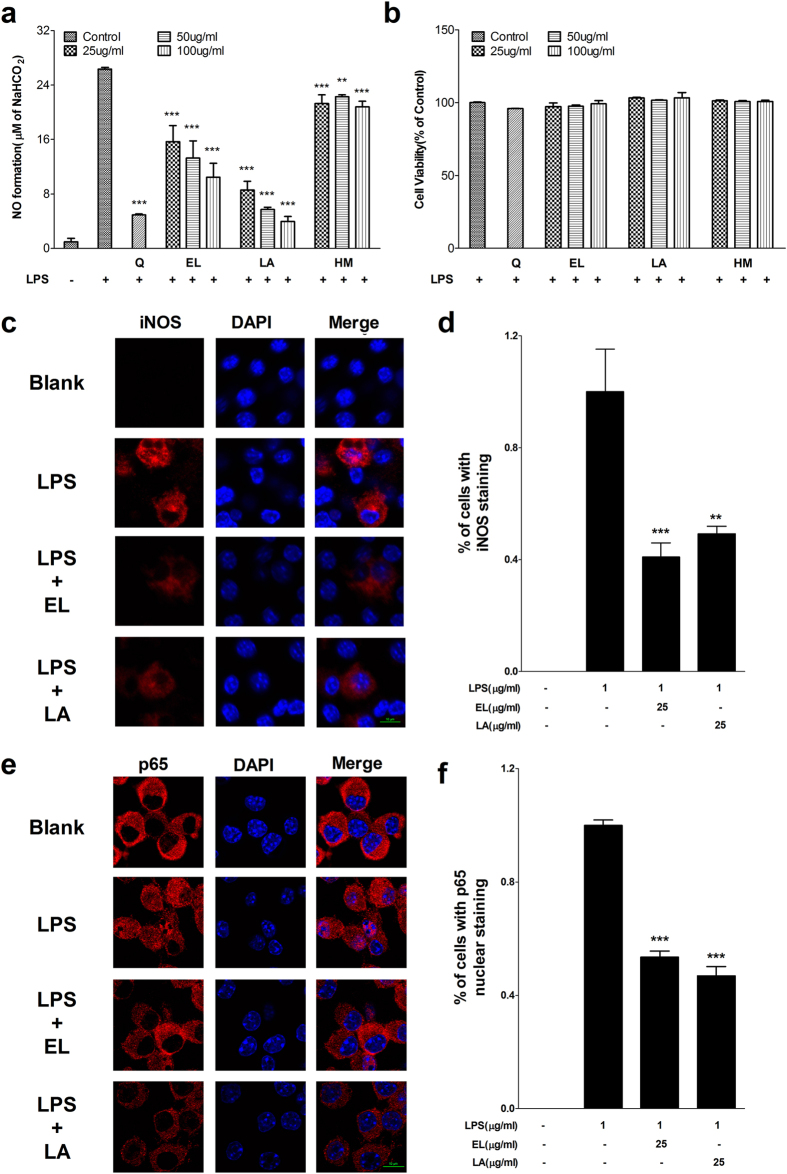
The anti-inflammatory activity of three compounds identified in MBF-DE. LPS (1 μg/ml) stimulated RAW 264.7 macrophage cells were treated with ethyl linolenate (EL), linoleic acid (LA) or Hydroxyl methylfurfural (HM) at concentrations indicated. Quercetin (Q) was used as the positive control. NO production (**a**), cell viability (**b**), iNOS expression (**c**) and p65 nuclear translocation (**d**) were determined as described in material and methods. Data represent means ± SEM; **P < 0.01, ***P < 0.001 compared to LPS control.

To identify the potential functional component in MBF-DE, seven pooled fractions via silica-gel column chromatography with cyclohexane-ethyl acetate gradient were collected, and their impact on NO production and cell viability were measured in LPS stimulated RAW264.7 cells. Fraction 2 (F2), F5, F6, and F7 had shown more profound bioactivity in inhibiting NO production than other fractions, without altering cell viability (Supplementary Fig. 2). Therefore, F2, F5 and F6 were further purified by HPLC to obtain compound 1 (C1), C2 and C3. By combining the GC-MS and NMR analysis, the structure of C1, C2 and C3 were elucidated as linoleic acid (LA), ethyl linolenate (EL) and hydroxyl methylfurfural (HM) (Supplementary Fig. 3), respectively. Further experimental results indicated that EL and LA rather than HM exhibited significant anti-inflammatory activity by inhibiting NO production, expression of iNOS and nuclear translocation of NF-κB p65 in LPS stimulated macrophage cells, respectively (Fig. 5). These results showed that LA and EA could inhibit LPS induced inflammatory responses.

### Dietary MBF ameliorated DSS-induced acute colitis

**Figure 6:**
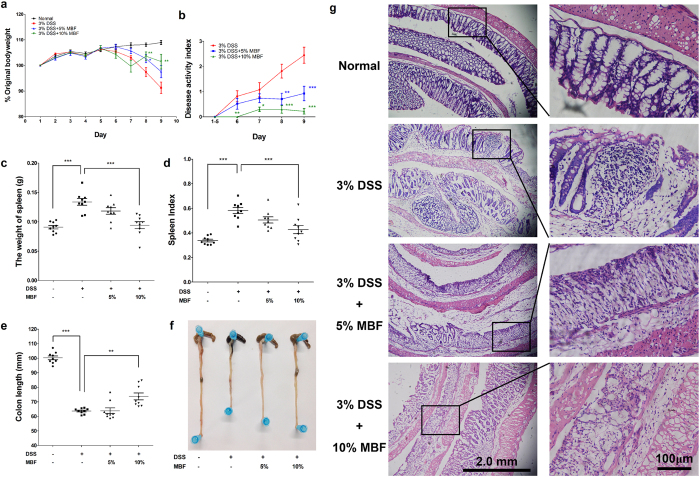
MBF dietary supplementation attenuates pathological symptoms of DSS induced acute colitis. BALB/c mice were fed with MBF (20 mg/kg) for 10 days prior to exposure to 3% DSS in drinking water. Daily weights were measured (n = 6–8/group) and plotted as percentage of body weight change from initial weight (**a**). Pathological parameters including disease activity index (**b**), weight of spleen (**c**), spleen index (**d**) and colon length from experimental mouse groups (**e,f**) were measured as described in methods. Colorectal histology changes (**f**) were determined by immunohistochemical staining. Data are representative of two independent experiments (means ± SEM). *P < 0.05, **P < 0.01, ***P < 0.001 compared to DSS-only control.

The therapeutic potential of MBF in suppressing acute colitis was investigated using the DSS induced mouse model. Compared to normal diet-fed mice, those fed with 3% DSS diet for 9 days appeared 10% loss in their initial body weight (Fig. 6a), 40% increase in spleen weight (Fig. 6c) and 35% shortening in colon length (Fig. 6d–f). However, these detrimental effects of DSS stimulation were significantly ameliorated by feeding mice with 5% or 10% MBF diet (Fig. 6a,c–f). Moreover, MBF diet significantly inhibited DSS induced disease activity index (Fig. 6b), a measurement of the production of bloody stools^31^. Likewise, results of histological analysis indicated that DSS induced severe injuries in colon crypts were significantly prevented by MBF dietary (Fig. 6g).

### Dietary MBF inhibited intestinal inflammation and tumorigenesis in MUC2^−/−^ mice

**Figure 7:**
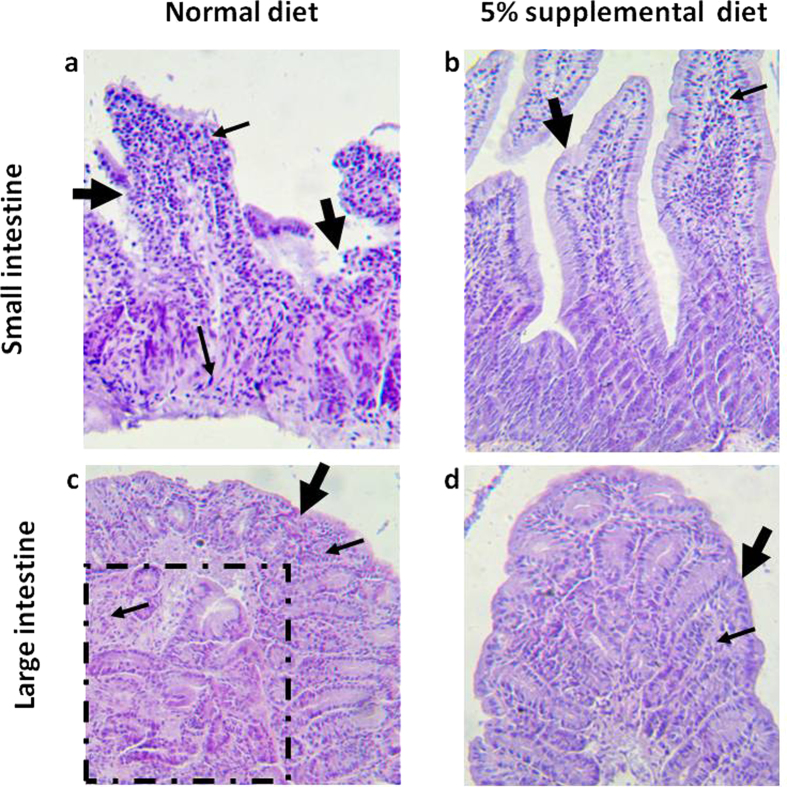
MBF dietary supplementation inhibited intestinal inflammation and tumorigenesis in Muc2^−/−^ mice. (**a**) several inflammation in small intestine. Small intestine showed large and continuous epithelium lesions, ulceration (large arrows) and intensive infiltrations of numerous inflammatory cells (small arrows) throughout the villus, mucosa, submucosa and even muscle layer of the intestinal wall; (**b**), MBF treated mouse small intestine showed virtually normal appearing mucosa and villus (large arrow) except for some inflammatory cell infiltration (small arrow); (**c**), Large intestine showed mucosa damage (large arrow) and tumor (dotted area) with inflammatory cells (small arrows) infiltration in the mucosa and tumor area; (**d**), MBF treated mouse large intestine showed virtually normal appearing mucosa (large arrow) except for few inflammatory cell infiltration (small arrow) in large intestine.

According to our previous reports^32^,^33^, the MUC2^−/−^ mouse spontaneously developed chronic intestinal inflammation at early age and progressed to intestinal tumors after 3 months. As shown in Table 1 and Fig. 7, 100% of the MUC2^−/−^ mice fed with normal diet developed intestinal tumors, but 5% or 10% dietary MBF significantly inhibited intestinal tumor formation, deceased tumor incidence to 20% and 30%, respectively. Consistent with the inhibition of tumor incidence, tumor numbers decreased from 2.3 ± 1.0 per mouse in normal diet groups to 0.2 ± 0.1 and 0.3 ± 0.1 tumors per mouse in 5% or 10% dietary MBF groups, respectively. Moreover, the dietary MBF also prevented intestinal inflammation. As shown in Fig. 7, the MUC2^−/−^ mice on normal rodent diet developed severe intestinal inflammation. The small intestine showed large and continuous epithelium damage, ulceration and intensive infiltrations of numerous inflammatory cells throughout the villus, mucosa, submucosa and even muscle layer of the intestinal wall (Fig. 7a). However, these lesions could be prevented by MBF supplementation, in which, the MBF-treated mouse small intestine showed virtually normal appearing mucosa and villus except for some inflammatory cell infiltration (Fig. 7b). Moreover, the large intestine of the normal dietary mice showed the development of tumor and inflammation, demonstrating the damage of mucosa and lymphocyte infiltration in the mucosa (Fig. 7c). In contrast, MBF treated mouse large intestine showed virtually normal appearing mucosa except for few inflammatory cell infiltration (Fig. 7d), similar as seen in the small intestine. Compared to 50% of normal diet-fed mice, only 10% and 20% of the mice fed with 5% or 10% dietary MBF developed intestinal inflammation.

## Discussion

In this study, we demonstrated that different MBF extracts, i.e., MBF-DE, MBF-EE, MBF-BE and MBF-WE, exhibited significant inhibitory activities on NO production without affecting cell viability in LPS stimulated macrophages (Fig. 1). Similar results have been reported in different extracts of other berry fruits including red raspberry, black raspberry and blackberry^31^. As a signaling molecule, NO can reflect the progress of inflammation while its over-production is mainly due to inducible nitric oxide synthase (iNOS) in response to inflammatory stimuli^34^. As expected, we found that LPS stimulation significantly induced the expression of iNOS, which in turn was suppressed by the treatment of MBF extracts at both mRNA and protein levels in the presence of LPS (Fig. 2). Besides the iNOS, we further showed that MBF extracts treatment significantly decreased LPS induced upregulation of COX-2, IL-1β and IL-6 (Fig. 2 and Supplementary Fig. 1). Since all these genes are recognized as important proinflammatory regulators^28^,^29^,^30^, it was strongly suggested that the different extracts of MBF (MBF-DE, MBF-EE and MBF-BE) inhibited the production of NO through suppressing the expression of proinflammatory genes^31^,^34^.

Notably, among different MBF extracts, MBF-DE showed a dose-dependent inhibition of LPS induced inflammatory reactions, i.e. higher concentrations of MBF-DE caused greater reduction in NO production and gene expression of the proinflammatory regulators (Fig. 2). These data suggested that the majority of active anti-inflammatory ingredients might exist in MBF-DE, and thus we decided to use MBF-DE in the subsequent experiments, in an attempt to identify the underlying mechanisms of anti-inflammatory effect and potent functional monomeric compounds.

**Figure 8:**
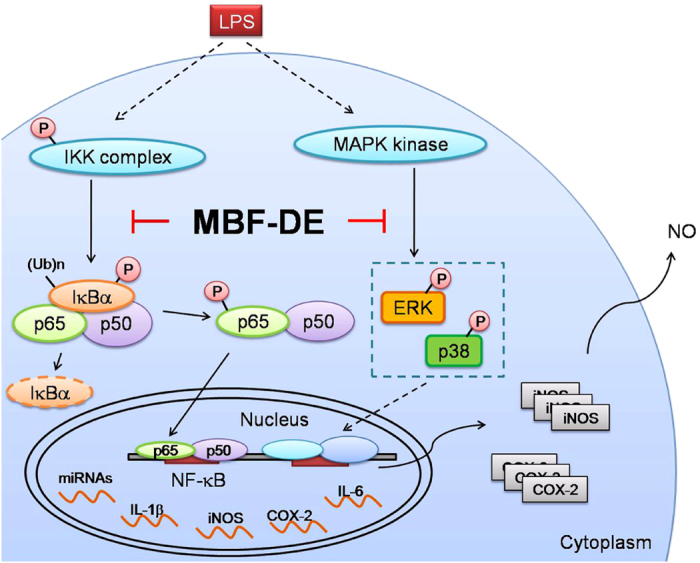
A schematic model that proposes the potential contribution of MBF in anti-inflammatory signaling pathways. LPS: lipopolysaccharide; MBF-DE: mulberry fruit dichloromethane extracts; NO: nitric oxide.

It has been well recognized that NF-κB is a key transcription factor that plays a pivotal role in the onset of inflammation and tumor progression^29^,^35^. In normal condition, functional NF-κB dimers are located in cytosol, combining with its inhibitor protein IκBα^35^. By contrast, in response to inflammatory stimuli, NF-κB was activated through the phosphorylation and subsequent degradation of IκBα/β by IKK complex^36^. The activated state of NF-κB dimer dissociates from IκBα/β in the cytosol and subsequently translocates to the nucleus, where it induces the expression of various inflammatory genes, including iNOS, COX-2 and TNF-α^37^,^38^,^39^. Similarly, MAPKs as a large family of seine/threonine kinases can also largely mediated the inflammatory signaling from the cell surface to the nucleus. Upon extracellular stimulation, these cytoplasmic enzymes are activated and thus they can modulate the activities of other intracellular proteins by adding phosphate groups to their serine/threonine amino acids. Ultimately, various transcription factors present in cytoplasm or nucleus could be phosphorylated and activated by three major groups of MAPKs, i.e., ERK1/2, p38 and JNK, leading to expression of different inflammatory mediators mentioned above^38^,^39^. In this work, both NF-κB and MAPK pathways were studied due to the strong inhibitory effects of MBF-DE on the expression of inflammatory genes (Fig. 2). Our study showed that MBF-DE inhibited the translocation of NF-κB p65 from cytoplasm to nucleus through blocking the LPS induced phosphorylation of IκBα (Figs 3 and 4), and suppressed the MAPK signals by suppressing the phosphorylation of ERK and p38 (Fig. 3). These findings suggested that MBF-DE treatment attenuated LPS induced inflammatory response, which could be attributable to the suppression of both NF-κB/p65 and MAPK/p38/ERK signals (Fig. 8). However, it should be noted that since the cell viability was not affected by the MBF-DE treatment (Fig. 1b), the inhibition of these two signals might not associated with the cell proliferation or apoptosis, although the exact molecular mechanism and functional target of MBF-DE need to be explored in future.

In this work, we were surprised to identify LA and EL, two long-chain unsaturated fatty acids (Supplementary Fig. 3), as the biofunctional monomeric compounds in MBF-DE (Fig. 5). It has been studied that the main chemical compositions of MBF were constituted by alkaloids, polyphenols, flavonoids and anthocyanins^13^,^14^, and most of which were reported to be responsible for its health benefits^9^,^10^,^11^. One possible explanation is that many other functional chemical components had been lost during the purification process, whereas long-chain unsaturated fatty acids, such as LA and EL, had been retained in the fraction (MBF-DE) that was used for further purification. Interestingly, we observed potent anti-inflammatory activity of LA and EL via suppression of NO production, gene expression of proinflammatory regulators and nuclear translocation of NF-κB p65 in LPS stimulated macrophages (Fig. 5), in a comparable manner to that of MBF-DE treatment (Fig. 4). Recent studies have shown high biofunctional activity of LA, a major fatty acid in MBF^13^, such as anti-hypertrophic^40^, anticancer^41^,^42^,^43^, anti-inflammatory^44^ and antioxidant bioactivities^45^,^46^. Therefore, we postulate that LA and EL may contribute to the anti-inflammatory activity of MBF observed in our study and previous studies. Nevertheless, further study is required to substantiate their bioactivities *in vivo* and identify more potentially functional compounds in MBF-DE.

For a long time, chemical induced or genetic engineering animal models have been widely used to study the prevention and diagnosis of IBD and related cancer^47^,^48^,^49^. For instance, DSS exposed mice have been commonly used as an acute colitis model that consistently recapitulates histopathological relevance to human disease^47^,^50^. While genetically deficient MUC2^−/−^ mice that spontaneously develop intestinal adenocarcinomas are often used as colorectal cancer model^25^,^32^. Based on the advance in research of these models, it is suggested a integration of pharmacologic and dietary interventions may have advantages of preventing the development of IBD and associated diseases^23^,^24^,^25^. In the present work, we demonstrated that dietary MBF resulted in a remarkable attenuation of clinical symptoms of acute colitis, such as body weight loss, colon shortening and histological damage (Fig. 6), and a significant reduction of intestinal inflammation and tumor formation in MUC2^−/−^ mice (Fig. 7). These findings implied potential preventative and therapeutic values of MBF in the management of acute colitis and colon cancer. However, it worth noting that these beneficial effects of the *in vivo* study using a MBF diet, which contain all kinds of biofunctional ingredients, could be very different from the *in vitro* study using MBF extracts by chemical reagents. It is common that some bioactive compounds tested in *in vitro* study fail to function *in vivo*. Moreover, recent studies have shown that exogenous plant elements such as miRNAs acquired through food intake could regulate the expression of their target genes in mammals, leading to a therapeutic effect on the corresponding diseases^51^,^52^. In addition, the change in the expression of p-p38 was measured by immunohistochemical staining in DSS mice model. We found that dietary MBF inhibited the expression of p-p38 in colonic tissue as compared with the DSS induced mice (Supplementary Fig. 4), suggesting the attenuation of DSS induced acute colitis might related to the suppression of p38/MAPK signal. Nevertheless, the specific dietary component with anti-inflammatory/anticancer activities and the underlying mechanisms *in vivo* remain to be elucidated in future work.

## Conclusion

We have demonstrated the *in vitro* and *in vivo* anti-inflammatory activity of MBF in RAW264.7 macrophages and DSS induced acute colitis mouse model, and its *in vivo* antitumor activity in MUC2^−/−^ mice model of colon cancer. We also provided further insights into the possible underlying mechanisms and therapeutic potency of MBF in intestine inflammation and associated cancer. The treatment with MBF extracts resulted in a significant decline in LPS-induced NO production that was associated with altered expression of iNOS, whereas the amelioration of inflammatory responses in LPS stimulated macrophages may be attributable to the downregulation of proinflammatory regulators, such as COX-2, IL-4 and IL-1β. The LA and EL are likely the major contributors to the strong anti-inflammatory activity of MBF-DE, which appeared to act by blocking NF-κB/p65 nuclear translocation and pERK/MAPK phosphorylation. MBF dietary showed therapeutic potency in the treatment of DSS induced acute colitis in mice and colon cancer in MUC2^−/−^ mice. Our results have demonstrated that MBF extracts are indeed involved in preventing intestinal inflammation and the associated tumorigenesis *in vitro* and *in vivo*, supporting the development of MBF as a functional food for testing in human clinical trials.

## Methods

### Chemical and reagents

Lipopolysaccharide and dextran sulfate sodium were purchased from Sigma (St. Louis, MO, USA). RPMI-1640 medium and fetal bovine serum were obtained from Hyclone (Logan, Utah, USA). Primary antibodies specifically against iNOS, COX-2, phospho-IKKα/β, IKKα, IKK β, phospho-IκBα, IκBα, p65, phospho-p65, phospho-p38, phospho-JNK, phospho-ERK, and β-tubulin were obtained from Cell Signaling Technology (Beverly, MA, USA). Anti-H3 and anti β-actin antibodies were purchased from Proteintech (Wuhan, China). HRP-conjugated goat anti-rabbit and goat-mouse secondary antibodies were provided by Beyotime (Shanghai, China). Cy3-conjugated anti-rabbit IgG secondary antibodies were purchased from Jackson ImmunoResearch. All other chemicals were of analytical grade from reputable suppliers.

### Preparation of MBF extracts and identification of single compound

Mulberry fruits (MBFs) at a commercial maturation stage were purchased from a local market of Shenzhen, Guangdong province. The procedure of MBF extracts preparation and single compound purification is shown in Supplementary Fig. 5. In brief, MBFs were homogenized in 80% ethanol containing 0.1N HCl by stirring at room temperature. The homogenate was filtered through a cotton filter and the residue was re-extracted for three times. Combined extracts were centrifuged at 8000 × g for 15 min, and the supernatant was concentrated using a vacuum rotary evaporator (EYELA). The resulting solid products were dissolved in water and then extracted with dichloromethane, ethyl acetate and n-butanol by agitating the suspension, respectively. The MBF water extracts (MBF-WE), dichloromethane extracts (MBF-DE), ethyl acetate extracts (MBF-EE) and n-butanol extracts (MBF-BE) were vacuum-dried, resuspended in dimethyl sulfoxide (DMSO), and stored at −80 °C before use. MBF-DE was further purified using silica-gel column chromatography with cyclohexane-ethyl acetate gradient and High Performance Liquid Chromatography (HPLC). The structures of identified single molecules were constructed by comparing the gas chromatography/mass spectrometry (GC/MS) and nuclear magnetic resonance (NMR) data with the authentic database.

### Cell experiments

The mouse RAW 264.7 macrophage cells (American Type Culture Collection) were routinely cultured^31^. In brief, cells were cultured in RPMI-1640 medium with 10% fetal bovine serum (Hyclone). Following subculture, cells were plated in 96-well plate for 2 h at a density of 1.0 × 10^5^ cells/well, and then treated with different MBF extracts (MBF-DE, MBF-EE, MBF-BE, MBF-WE) at the concentration range of 25–200 μg/ml diluted by culture medium in the absence or presence of 1 μg/ml LPS for 24 h. NO levels in the culture media were determined by Griess Reagent System according to manufacturer’s instructions (Promega). Cell viability was measured using the CellTiter 96 Aqueous One Solution Proliferation Assay Kit (Promega). NF-κB/p65 and iNOS immunofluorescence analysis were determined as described previously^31^. Quercetin (Sigma) was used as the positive control and the final concentration was 12.5 μg/ml. Control cells were grown under identical conditions without the test extracts.

### Real-time quantitative PCR

The total RNA was isolated using RNAiso Plus (TaKaRa Biotechnology) according to the manufacturer’s instructions. The concentration of RNA was quantified using the NanoDrop 2000c Spectrophotometer (Thermo Fisher Scientific). The first strand cDNA was synthesized from 1 μg of DNase treated total RNA using oligo(dT)_18_ plus random hexamer primers and M-MLV Reverse Transcriptase (Takara Biotechnology). The qPCR experiments were conducted on Step-One plus real-time PCR System (Applied Biosystems) using SYBR green-I Master PCR Mix with gene specific primers (Supplementary Table 1), and RPL14 was used as reference gene for normalization. The amplifications were performed on three independent samples and triplicate reactions were carried out for each sample. The relative mRNA level was calculated using the 2^−ΔΔCT^ method.

### Western blotting

Protein extraction and western blotting assay were performed as described previously^31^. In brief, total protein was extracted with RIPA lysis buffer supplemented with protease inhibitor cocktail (Roche). Protein level of samples was measured using a BCA protein assay kit (Thermo Fisher Scientific, Rockford, IL, USA). Equal protein amounts (30 μg) were loaded for 10% sodium dodecyl sulphate-polyacrylamide gel electrophoresis (SDS-PAGE) and transferred to a polyvinylidene difluoride (PVDF) or nitrocellulose membrane. After blocking the nonspecific site with 5% non-fatted milk for 1 h, the membrane was incubated with specific primary antibody overnight at 4 °C, and then incubated with horseradish peroxidase conjugated secondary antibody for 1 h at room temperature. The immune-blotting signals were visualized with ECL kit (West-Pico, Super Signal; Pierce, Rockford, IL, USA) and exposed to X-ray film. Each protein band was quantified using Carestream Molecular Imaging Software (Carestream Health, Toronto, Canada).

### Enzyme-linked immunosorbent assay (*ELISA*) measurement

The cell culture medium were centrifuged at 8000 rpm for 10 min at 4 °C and the cell-free supernatants were collected for the determination of IL-1β and IL-6 using commercial mouse ELISA kits (4Abio., Beijing, China) according to the manufacturer’s instructions. The OD of the microplate was read at 450 nm.

### Animal disease models and diets

All animal experiments were approved by the Animal Ethical and Welfare Committee of Shenzhen University (no. AEWC-2014 -001314). All procedures involved in the animal experiments were carried out in accordance with the approved guidelines and regulations.

### 1) Dextran sulfate sodium (DSS)-induced acute colitis

DSS induced acute colitis model has been described previously^31^. Six- to eight-week-old BALB/c mice were fed with either commercial mouse food as control group or the same food supplemented with 5% or 10% MBF powder for 10 days prior to exposure to 3% DSS in drinking water for 9 days. The pathological and histological parameters including body weight loss, weight of spleen and colon length were measured as previously described^31^.

### 2) MUC2^−/−^ mouse model

The MUC2^−/−^ mouse model and the methods for genotyping have been reported previously^32^,^53^,^54^. After weaning (approximately 3–4 weeks after birth), littermates were randomized to dietary groups and fed *ad libitum* either with normal rodent diet or the normal diet supplemented with 5% or 10% MBF powder. At the end of feeding for 3 months, all animals were sacrificed and tumor incidence, frequency and histopathology were evaluated, as described previously^25^,^33^. As to the evaluation of intestinal inflammation, we adopted the histopathological inflammation score criteria published recently by us^55^. The degree of the intestinal inflammation was based on the degree of intestinal mucosa damage (including epithelial lesion, ulceration, etc) and lymphocyte infiltration (including the numbers of infiltrated inflammatory cells and the depth of the inflammatory cell infiltrated, such as to the mucosa, submucosa or the entire wall of the intestine.) The comparison was made between the small intestine and large intestine of the Muc2^−/−^ mice groups fed with normal rodent diet or MBF-supplemented diet, respectively.

### Statistical analysis

Statistical analyses were performed with SPSS version 19.0 software 261 package (SPSS, Chicago, IL, USA). Data are presented as mean ± SD (standard deviation) or mean ± SE (standard error) of three independent experiments. Significant differences between the groups were determined using a one-way analysis of variance (ANOVA), considering *P < 0.05, **P < 0.01, ***P < 0.001 as significant differences.

## Additional Information

**How to cite this article**: Qian, Z. *et al*. Mulberry fruit prevents LPS-induced NF-κB/pERK/MAPK signals in macrophages and suppresses acute colitis and colorectal tumorigenesis in mice. *Sci. Rep*. **5**, 17348; doi: 10.1038/srep17348 (2015).

## Supplementary Material

Supplementary Information

## Figures and Tables

**Table 1:** MBF dietary supplementation inhibits intestinal inflammation and tumorigenesis in MUC2^−/−^ mice.

	Intestinal tumor			Intestinal inflammation		
	Incidence(%)	Number (Mean +/−SD)	p value	Incidence(%)	Number (Mean+/−SD)	p value
Normal diet	100%(10/10)	2.3+/−1.0		50%(5/10)	0.7+/−0.8	
5%MBF	20%(2/10)	0.2+/−0.1	<0.001	10%(1/10)	0.1+/−0.3	<0.05
10%MBF	30%(3/10)	0.3+/−0.1	<0.001	20%(2/10)	0.2+/−0.1	0.1
