# Supplementary material for: Mulberry fruit prevents LPS-induced NF-κB/pERK/MAPK signals in macrophages and suppresses acute colitis and colorectal tumorigenesis in mice
Source: Sci Rep. 2015 Nov 30;5:17348. doi: 10.1038/srep17348 (PMC4663626; doi:10.1038/srep17348)
Supplement: Supplementary Information [file srep17348-s1.doc]

# Mulberry fruit prevents LPS-induced NF-κB/pERK/MAPK signals in macrophages and suppresses acute colitis and colorectal tumorigenesis in mice

Zhengjiang Qian^1,2*^, Zhiqin Wu^1*^, Lian Huang^1^, Huiling Qiu^1^, Liyan Wang^1^, Li Li^1^, Lijun Yao^1^, Kang Kang^3^, Junle Qu^2^, Yonghou Wu^4^, Jun Luo^4^, Johnson J Liu^5^, Yi Yang^3^, Wancai Yang^6#^, Deming Gou^1#^

**Supplementary Information**

**Supplementary Figure 1 Effect of MBF extracts on the protein expression of IL-1β and IL-6 in LPS-stimulated RAW 264.7 macrophage cells**. Cells were stimulated for 6h with LPS (1μg/mL) alone or together with MBF-DE, MBF-BE and MBF-EE at concentrations indicated. Quercetin (Q) was used as a positive control. The protein expression of IL-1β (a) and IL-6 (b) were determined by ELISA assays. Results are shown as means ± SD of three independent experiments. * *P*<0.05, ***P*<0.01, ****P*<0.001 compared to LPS-induced control.

**Supplementary Figure 2 Effects of seven pooled fractions from MBF-DE on the NO production and cell viability in LPS stimulated RAW 264.7 macrophage cells.** Cells were treated with seven fractions of MBF-DE at different concentrations range from 50 to 100 μg/mL in the presence or absence of LPS (1 μg/ml) for 24h. NO production (a) and cell viability (b) were determined by the Griess and MTS assay, respectively. Data are expressed as means ± SD with at least three independent experiments.

**Supplementary Figure 3** **The structure of three indentified compounds in MBF-DE**. The structure of the identified single molecular was obtained by comparing the gas chromatography/mass spectrometry (GC/MS) and nuclear magnetic resonance (NMR) data with the authentic database.

**Supplementary Figure 4 Effect of MBF dietary supplementation on the expression of phospho-p38 in colonic tissue of DSS induced mice.** Representative sections of the immunohistochemical staining level of p-p38 expression in colonic tissue of normal (a), 3% DSS model (b), 5% MBF + 3% DSS (c) and 10% MBF + 3% DSS (d) diet mice.

**Supplementary Figure 5** **Procedure of MBF extracts preparation, purification and compound identification.** EtOH: ethyl alcohol; CHX: cyclohexane; EAC: [ethyl](javascript:void(0);) [acetate](javascript:void(0);); MeOH: [methyl](javascript:void(0);) [alcohol](javascript:void(0);); HPLC: High Performance Liquid Chromatography**.**


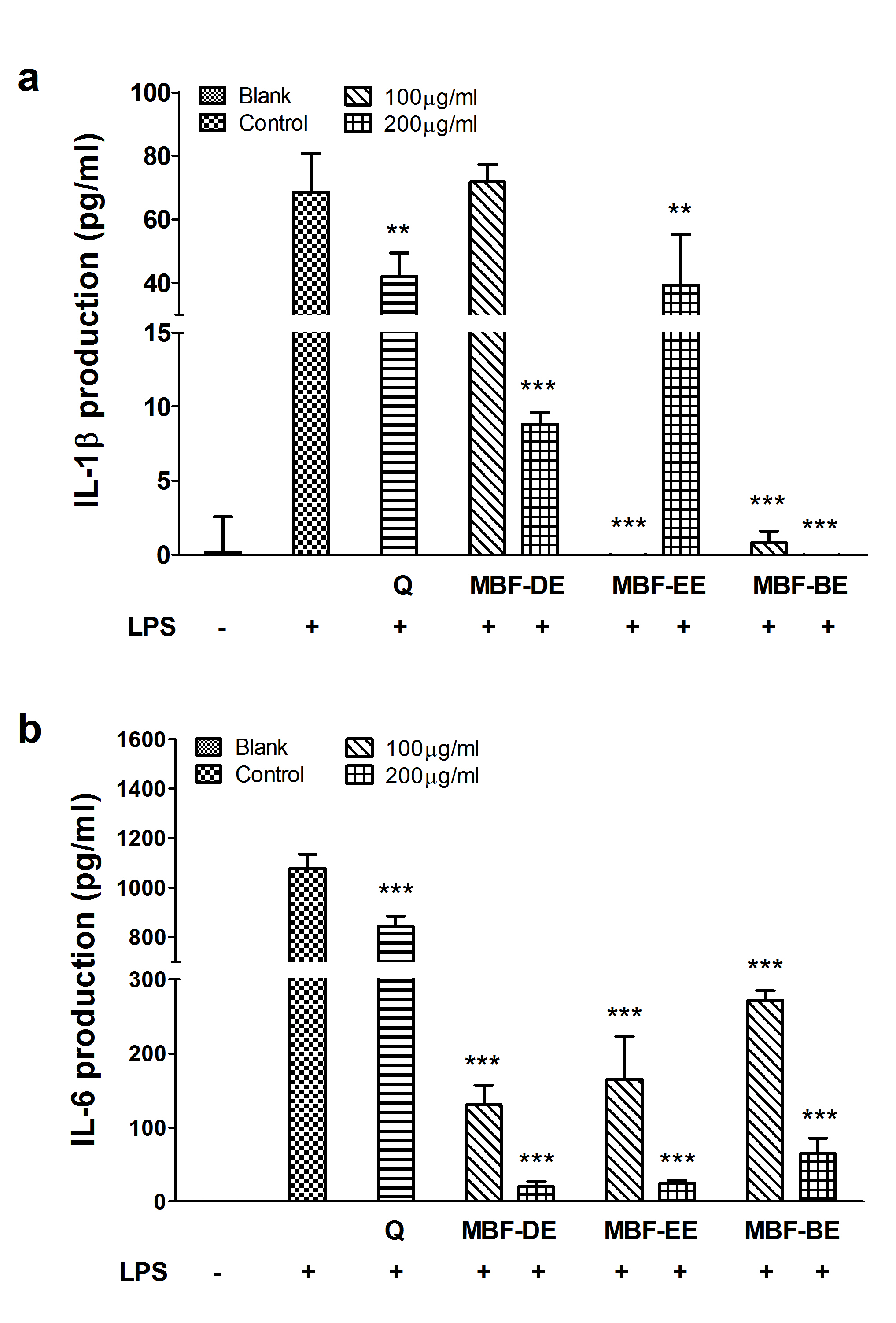


**Supplementary Figure 1**

**
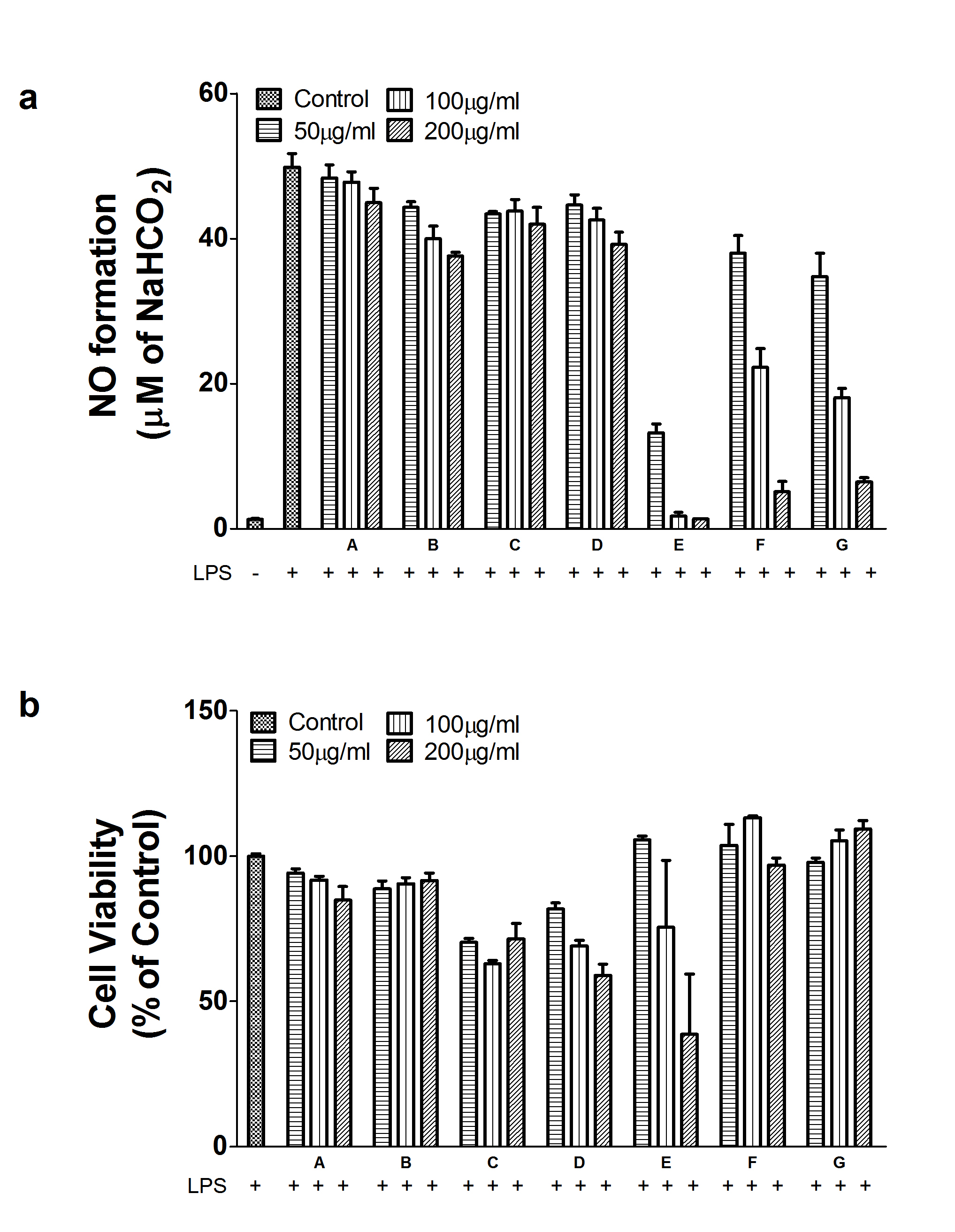
**

**Supplementary Figure 2**

**Supplementary Figure 3**

**
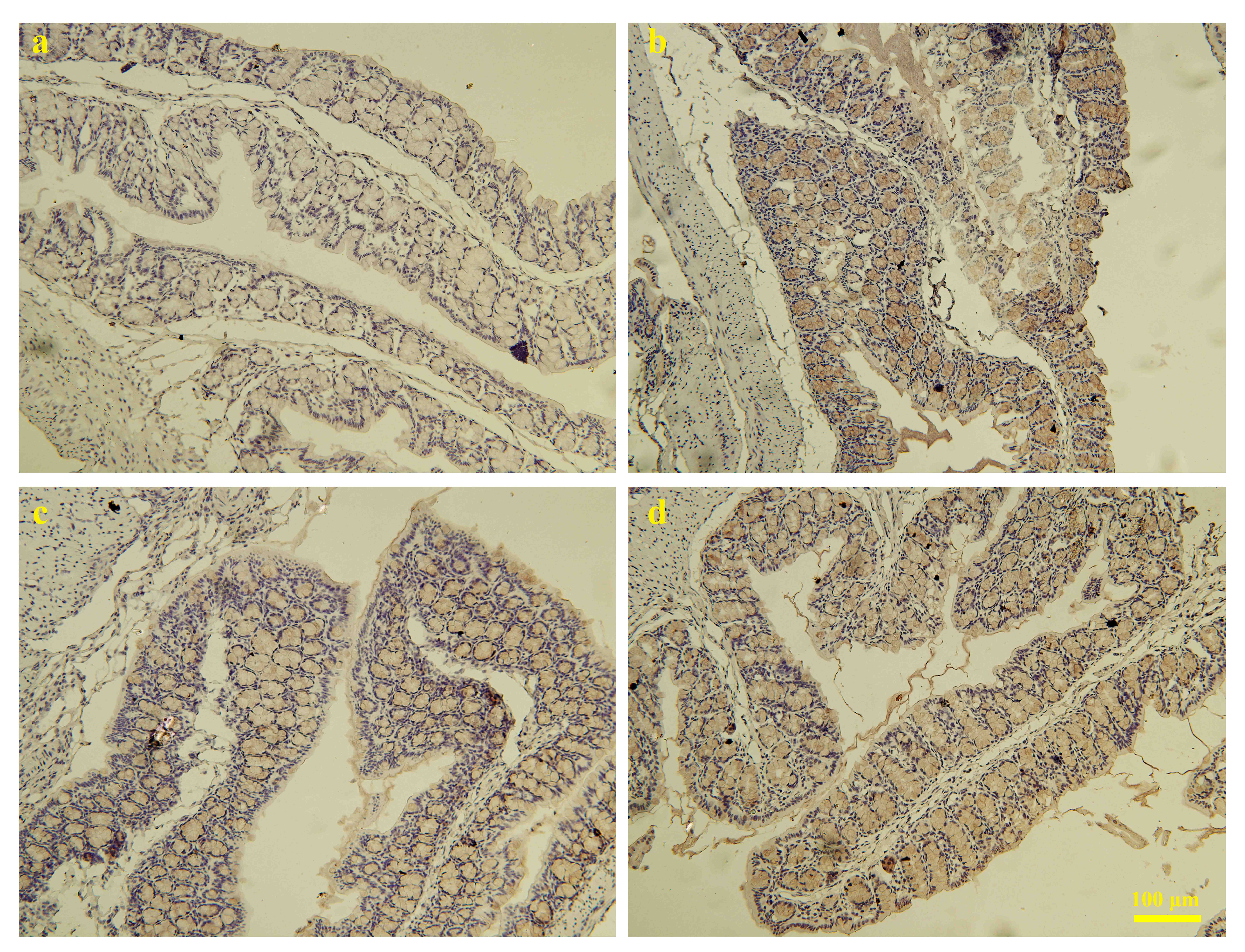
**

**Supplementary Figure 4**

**Supplementary Figure 5**

**Supplementary Table 1** Sequence of gene-specific primers used for real-time RT-PCR amplification.
